# The Foreign Language Effect on Moral Judgment: The Role of Emotions and Norms

**DOI:** 10.1371/journal.pone.0131529

**Published:** 2015-07-15

**Authors:** Janet Geipel, Constantinos Hadjichristidis, Luca Surian

**Affiliations:** 1 Department of Psychology and Cognitive Sciences, University of Trento, Trento, Italy; 2 Department of Management and Economics, University of Trento, Trento, Italy; 3 Research Centre for Decision Making, Leeds Business School, Leeds University, Leeds, United Kingdom; Middlesex University London, UNITED KINGDOM

## Abstract

We investigated whether and why the use of a foreign language influences moral judgment. We studied the trolley and footbridge dilemmas, which propose an action that involves killing one individual to save five. In line with prior work, the use of a foreign language increased the endorsement of such consequentialist actions for the footbridge dilemma, but not for the trolley dilemma. But contrary to recent theorizing, this effect was not driven by an attenuation of emotions. An attenuation of emotions was found in *both* dilemmas, and it did not mediate the foreign language effect on moral judgment. An examination of additional scenarios revealed that foreign language influenced moral judgment when the proposed action involved a social or moral norm violation. We propose that foreign language influences moral judgment by reducing access to normative knowledge.

## Introduction

The capacity to deliver moral judgments is a core aspect of social competence in humans. Philosophical and psychological investigations of moral cognition have often focused on complex dilemmas that create a tension between a deontological mode of responding, which stresses adherence to moral rules (e.g., “do not kill or harm innocent people”), and a consequentialist mode, which aims at maximizing the anticipated outcome (e.g., “do the greatest good for the greater number of individuals”). Research has shown that some moral scenarios tend to promote consequentialist judgments, while other scenarios, typically those that involve personal force and the instrumental use of a person [1, 2], tend to promote deontological judgments. We follow Greene [3] in using deontological and consequentialist to mean “characteristically deontological” and “characteristically consequentialist” as a function of response content, not the underlying motivation. For example, in the well-known trolley problems [4, 5], most people judge that it is acceptable to save five persons by hitting a switch that would produce one collateral victim (standard trolley problem), but deem it unacceptable to save five persons by pushing a person off a bridge (footbridge problem) [6, 7]. Why do people accept to save the lives of five by hitting a switch but not by pushing a person?

Two main kinds of explanations have been proposed. One emphasizes the causal structure of actions and their contexts and posits the effect of tacit knowledge of abstract moral principles [8–10]. The other perspective emphasizes the selective activation of different processing routes to moral judgment [2, 11, 12]. In this perspective, responses result either from one route that is characterized by automatic emotional processes or an alternative route that consists of controlled cognitive processes. Emotional, “heart” thinking prompts a deontological response, whereas deliberate, “head” thinking privileges a consequentialist response. Deontological responses will dominate when moral dilemmas trigger a strong emotional response (e.g., footbridge problem), whereas consequentialist responses will surface when dilemmas are low in emotional salience (e.g., trolley problem).

Consistent with this view, priming the emotional system has been shown to privilege deontological responses, whereas priming the analytic system has been shown to preferentially support consequentialist choices [13,14]. Furthermore, cognitive load has been shown to selectively interfere with consequentialist responses, suggesting that such answers are products of controlled cognitive processes [15]. Additional evidence for this dual-process model of morality comes from neuropsychological studies, which show that brain-damaged patients with emotional deficits are more likely to give consequentialist responses to highly emotional dilemmas than controls [16–18].

Dual-process models of moral judgment may also guide research on language effects. In a recent study, Costa and colleagues reported that foreign language promotes consequentialist responses [19, 20]. These authors presented participants with the standard trolley and footbridge dilemmas either in a participants’ native language or in a foreign language. They recruited participants from several cultures and examined an impressive number of native—foreign language combinations. Foreign language systematically increased the rate of consequentialist responses for the footbridge dilemma but had no influence on the responses to the standard trolley dilemma. Following dual-process models of morality, these authors argued that foreign language influences moral choice by triggering cognitive and emotional distance, that is, by prompting cold, “head” thinking. Its effects are felt in the footbridge dilemma as this presumably triggers the “hot”, emotional system, but not in the trolley dilemma, which is presumably underpinned by the “cool”, controlled system. The authors supported their claim that foreign language attenuates emotions by referring to experimental evidence from bilingual studies [21, 22].

Here we address whether and why presenting moral dilemmas in a foreign language rather than the native language influences moral judgment. By foreign language we mean a non-native language that has been learned in a classroom context, that is, outside the environment where it is commonly used by native language speakers [23]. Our aim is twofold. First, we attempted to consolidate the findings of previous studies [19], which reported an increase of consequentialist responses in the footbridge dilemma, but not in the trolley dilemma. To this end, we presented these two dilemmas to native Italian speakers who learned either German or English as a foreign language (Study 1), and to native Chinese speakers who learned English as a foreign language (Study 2). Second, we aimed to investigate the claim that the foreign language effect on moral judgment is driven by reduced emotionality. For this purpose, alongside moral judgments we also gathered emotion ratings (Study 2). As a further test, we examined moral evaluations of additional high-emotion and low-emotion dilemmas (Study 3). If the effect is driven by reduced emotionality, then it should be more pronounced in the high-emotion dilemmas.

## Study 1

In Study 1, we tested the foreign language effect on moral judgment using the footbridge and trolley dilemmas. We recruited students enrolled at foreign language courses at the University of Trento, because we wanted to ensure a good understanding of the materials. One group received the dilemmas in their native language, Italian, whereas two other groups in a foreign language, either English or German. We tested two foreign languages to assess the generality of the foreign language effect.

### Methods

The study protocol was approved by the Ethics Committee of the University of Trento according to the principles expressed in the Declaration of Helsinki. We obtained participants’ informed verbal consent by using a verbal consent protocol. The Ethics Committee of the University of Trento waived the requirement of written consent forms.

#### Participants

Sample size was determined by conducting an a–priori sample size calculation using G*power [24] for a 2 × 2 χ2 test. The parameters were set as follows: effect size w = 0.4 (medium-high, estimated), alpha level = .05, power = .8, and degrees of freedom = 1. The calculation indicated a minimum sample size of 50. We tested more participants than the power analysis suggested because the present studies were conducted during classes in which a greater number of participants was available (this applies to all reported studies). No interim analyses or stopping rules were applied.

One hundred five students (88 female, 17 male; *M*_age_ = 22.08 years, age range: 19–46 years) volunteered to participate at the beginning of foreign language classes in German or English at the University of Trento. Thirty nine participants were randomly assigned to the native language condition (NL; Italian), 37 to the foreign language English condition (FL_English_), and 29 to the foreign language German condition (FL_German_). All participants had an *intermediate* level certificate (B = *independent user*) in the respective foreign language as specified by the *Common European Framework of Reference for Languages*: *Learning*, *Teaching*, *Assessment* (CEFR; [25]). On average, participants in the FL_English_ condition had English education since the age of 10.72, CI [8.62, 13.31], and those in the FL_German_ condition had German education since the age of 13.36, CI [11.74, 14.96]. Participants in the foreign language conditions were asked to self-assess their foreign language proficiency in terms of *conversational fluency*, *reading*, *writing*, and *understanding* on a 5-point scale (1 = *almost none*, 2 = *poor*, 3 = *fair*, 4 = *good*, 5 = *very good*; scale adapted from [26]). Across the four measures, the participants rated their foreign language skills between *fair* and *good* (FL_English_: *M* = 3.33, CI [3.17, 3.50], FL_German_: *M* = 3.79, CI [3.64, 3.95]).

#### Materials and procedure

We presented participants with the trolley and the footbridge dilemmas, together with a non-moral filler dilemma (all items were adapted from [2]; see Appendix A for the full text). Each moral dilemma stated an action (i.e., hitting a switch, pushing a person off a bridge) that would harm an individual but as a consequence save five persons. Participants had to judge the appropriateness of the proposed action by selecting *Yes* (consequentialist response) or *No* (deontological response). The filler item concerned a choice between travelling by bus or train given certain time constraints, and was designed to induce a high rate of endorsements. We expected no language effect for this item. Its purpose was to assess whether participants in the foreign language condition understood the materials–misunderstandings should drive the endorsement rate towards 50%.

The presentation order of the moral dilemmas was counterbalanced. In each condition, participants received a questionnaire entirely written in one language: Italian, English, or German. In all our studies, the original materials were in English, and were translated to other languages by bilinguals. Two independent judges controlled the translated versions for consistency with the English version. The language versions were also closely matched for word count.

### Results

**Fig 1:**
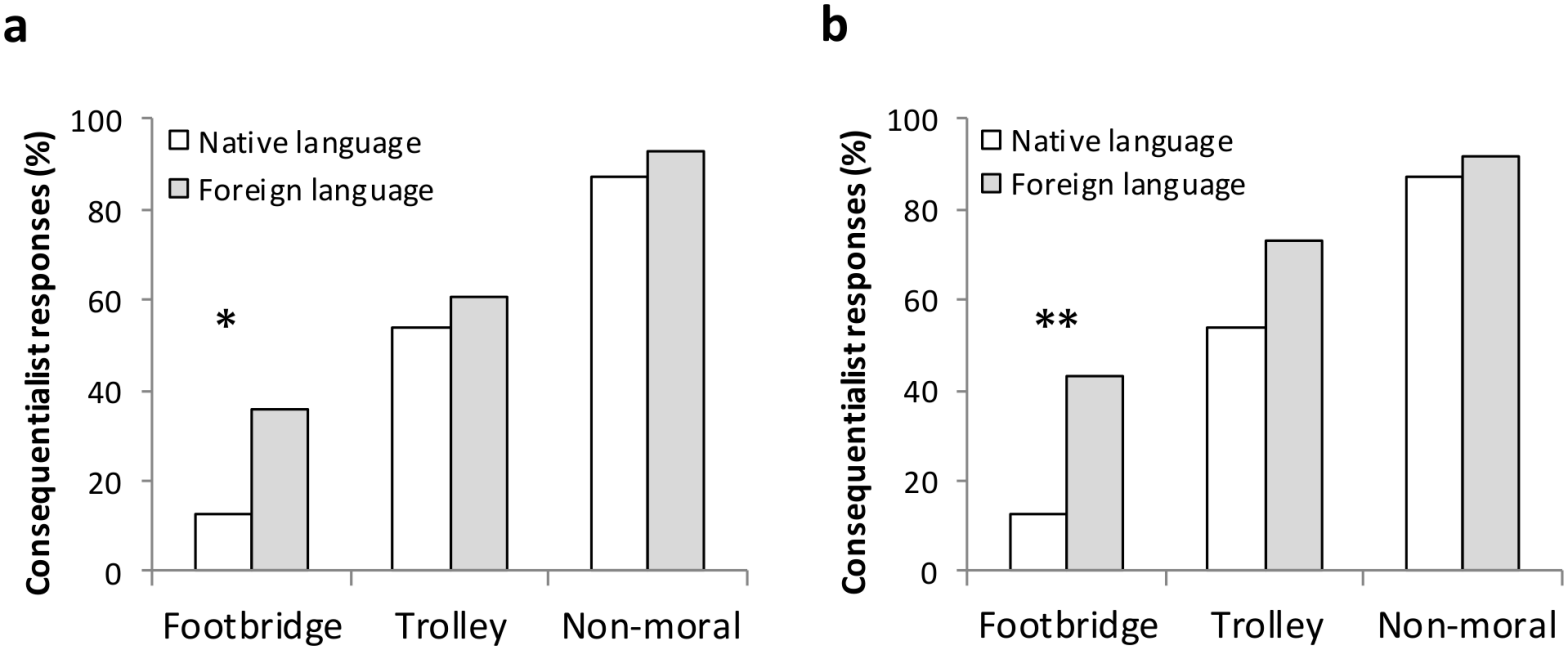
Percentage of consequentialist choices in Study 1. Percentage of participants who endorsed the consequentialist action, by dilemma type and language condition. The native language was Italian. The foreign language was either German (a) or English (b). *p < .05, **p < .01.

Preliminary analyses revealed no effect of presentation order. Hence, we dropped this factor from subsequent analyses. The main findings are illustrated in Fig 1. As anticipated, the use of a foreign language increased the rate of consequentialist responses in the footbridge dilemma but not in the trolley dilemma. While 12.8% of participants stated that it was appropriate to push the man off the footbridge when the dilemma was presented in the native language, this rate increased to 35.7% when it was presented in German, χ^2^ (1, *N* = 67) = 4.92, *p* = .027, φ = .27, to 43.2% when the dilemma was presented in English, χ^2^ (1, *N* = 76) = 8.79, *p* = .003, φ = .34, and to 40% if we collapse over foreign language condition, χ^2^ (1, *N* = 104) = 8.61, *p* = .003, φ = .29. In the trolley dilemma, 53.8% of participants chose to hit the switch when the dilemma was presented in the native language. Similar rates were observed when the language was German (60.7%), χ^2^ (1, *N* = 67) = 0.31, *p* = .576, φ = .07, and English (72%), χ^2^ (1, *N* = 76) = 2.99, *p* = .084, φ = .20, or the two pooled together (67.7%), χ^2^ (1, *N* = 104) = 1.99, *p* = .158, φ = .14.

In accord with previous research, within each language condition a higher proportion of participants endorsed the action of the trolley dilemma than of the footbridge dilemma (*p*s < .005, by binomial tests). Importantly, we observed no language differences for the non-moral filler item: FL_English_: 92.0% vs. NL: 87.2%, χ^2^ (1, *N* = 76) = .45, *p* = .50; FL_German_: 93.1% vs. NL: 87.2%, χ^2^ (1, *N* = 68) = 0.63, *p* = .43, φ = 0.10; FL_Total_: 92.4% vs. NL: 87.2%, χ^2^ (1, *N* = 105) = 0.78, *p* = .38, φ = 0.09. This suggests that the participants understood the materials.

#### Correlations between proficiency and moral judgment

We created a proficiency score by aggregating a participant’s self-ratings in reading and understanding (each scale ranged from 1 = *almost none*, to 5 = *very good*). We only considered these scales because they are the most pertinent for the current task. The highest possible score is 10, which we also assigned to the participants in the native language condition. In the footbridge dilemma, we found that the lower the language proficiency, the higher the rate of action endorsements: *r*(102) = –.22, *p* = .023. No correlation between proficiency and action endorsements were observed in the trolley dilemma, *r*(102) = –.09, *p* = .369, or the non-moral dilemma, *r*(102) = –.07, *p* = .505.

### Discussion

In line with prior research [19, 20], Study 1 showed that foreign language increases the rate of consequentialist responses in the footbridge dilemma but not in the trolley dilemma. This effect was robust across two foreign languages, English and German. Importantly, the use of a foreign language had no influence on the evaluation of a non-moral dilemma, which suggests that the effect is “real” and not due to misunderstanding.

## Study 2

In Study 2, we aimed to generalize the foreign language effect to a sample of native Chinese speakers who learned English as a foreign language. Although China accounts for roughly one fifth of the world’s population, relatively few studies have examined how Chinese respond to moral dilemmas. Some cross cultural studies found cultural differences [27], whereas others did not [28]. Relevant to the present study, previous research has shown that the reduction of emotional force in a second language is also observed in late Chinese–English bilinguals [29].

### Methods

#### Participants

In Study 2, we used a binary (*Yes/No*) measure and a more sensitive 7-point scale (see Materials and Procedure). To determine the appropriate sample size, we conducted two a–priori sample size calculations using G*power [24]. The first was for a 2 × 2 χ^2^ test, with the same settings as in Study 1. The calculations revealed a minimum sample size of 50. The second was for a 2 × 3 mixed ANOVA. The parameters were set as follows: effect size *f* = 0.3 (medium-high), alpha level = .05, power = .8, correlation among measures at .4. The calculation indicated a minimum sample size of 56. No interim analysis or stopping rules were applied.

We were granted access to courses in Tsinghua University, Wuhan University, and the Shanghai University of Sport, and tested 161 students (72 female, 88 male,1 unknown, *M*_age_ = 23.41 years, age range: 18–40). Ninety-nine participants were randomly assigned to the foreign language condition (English), and 62 to the native language condition (Chinese). All participants reported to have a Band–4 College English Test certification (CET-4), which is the standard *English as a foreign language* test administered in China. In addition, participants had to self-assess their language proficiency in English in terms of conversational fluency, reading, writing, and understanding on a 5-point scale (1 = *almost none*, 2 = *poor*, 3 = *fair*, 4 = *good*, 5 = *very good*). Averaging across the four measures (Cronbach’s α = .86) participants judged their English skills as *fair* (*M* = 3.15, CI [3.01, 3.29]). As a last task, participants assigned to the foreign language condition had to indicate whether they understood the scenarios on a 7-point scale (1 = *not at all*, 4 = *average*, 7 = *very well*; this question was presented in Chinese). We excluded nine participants who rated their understanding as 3 or less (their exclusion does not influence the main pattern of results). The data we report are from the remaining 152 participants.

#### Materials and procedure

For the moral judgment task, we used similar materials and procedure as in Study 1. Participants were presented with the footbridge and trolley dilemmas, separated by a non-moral filler item. The presentation order of the moral dilemmas was counterbalanced. Following each dilemma, participants evaluated the moral permissibility of the proposed action on a binary scale (*Yes*/*No*), but here also on a more sensitive 7-point scale (1 = *forbidden*, 4 = *permissible*, 7 = *obligatory*; [6]). After the moral judgment questions, participants were asked to rate the extent to which each dilemma made them feel distressed (*Thinking about the scenario I just read*, *I felt… upset*, *worried*, and *sad*). For each emotion, participants had to respond using a 7-point scale (1 = *not at all*, 4 = *somewhat*, 7 = *very much*; adapted from [30]).

### Results

Preliminary analyses revealed no effect of presentation order. Therefore, we dropped this factor from subsequent analyses.

#### Moral judgment (Yes/No)

**Fig 2:**
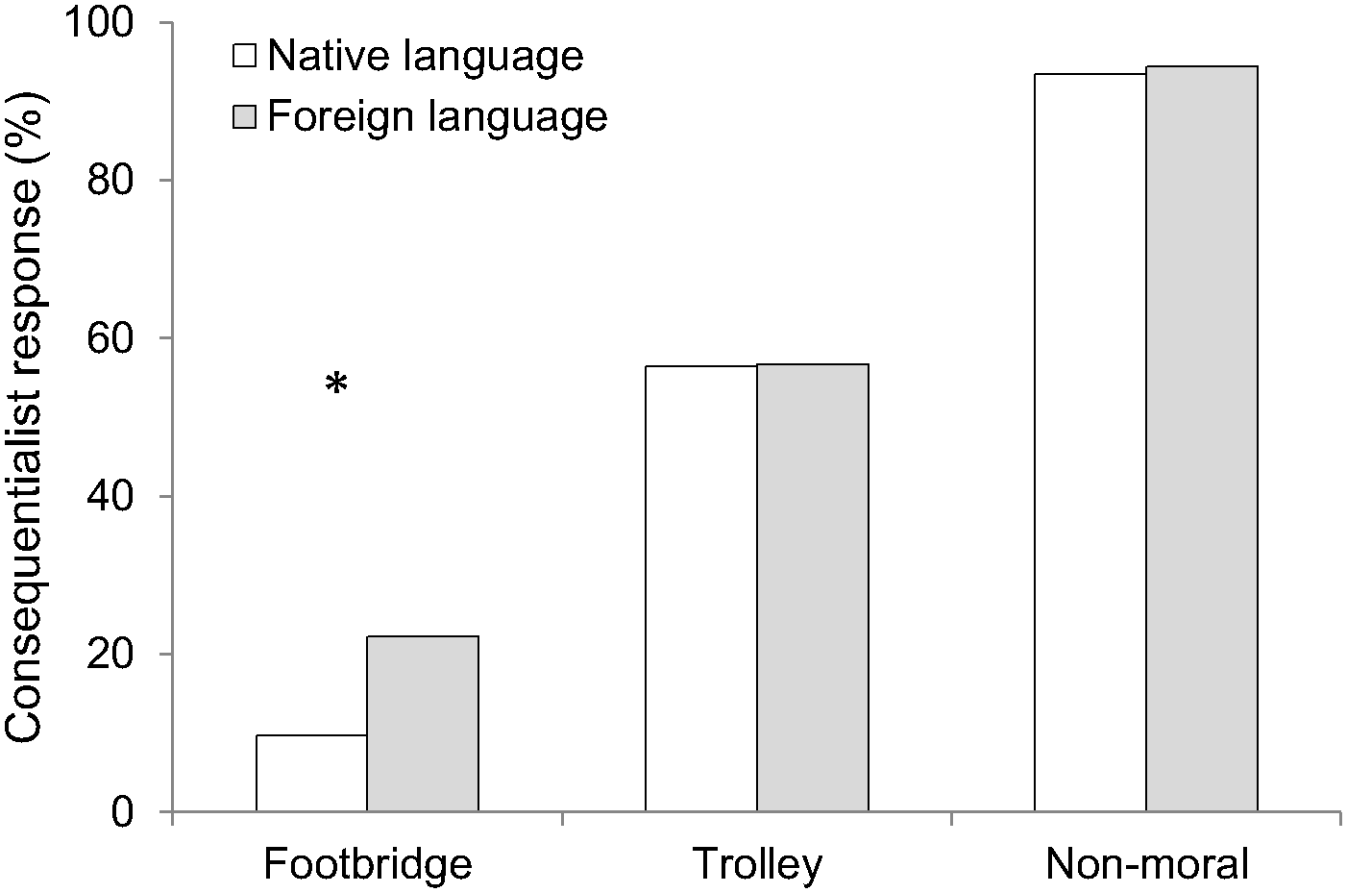
Percentage of consequentialist choices in Study 2. Percentage of participants who endorsed the consequentialist action, by moral dilemma and language condition (native language = Chinese, foreign language = English). *p < .05.

The main findings are illustrated in Fig 2. There was a significant foreign language effect for the footbridge dilemma (FL: 22.2% vs. NL: 9.7%), χ^2^ (1, *N* = 152) = 4.07, *p* = .044, φ = .16, but not for the trolley dilemma (FL: 56.7% vs. NL: 56.5%), χ^2^ (1, *N* = 152) < 1, *p* = .979, φ < .01. In line with Study 1 and previous research, within each language condition, a higher proportion of participants endorsed the action of the trolley versus the footbridge dilemma (*p*s < .001, by binomial tests). No language effect was present for the non-moral item (FL: 94.4% vs. NL: 93.5%), χ^2^ (1, *N* = 152) < 1, *p* = .818, φ = .02.

#### Moral judgment (7-point Scale)

**Fig 3:**
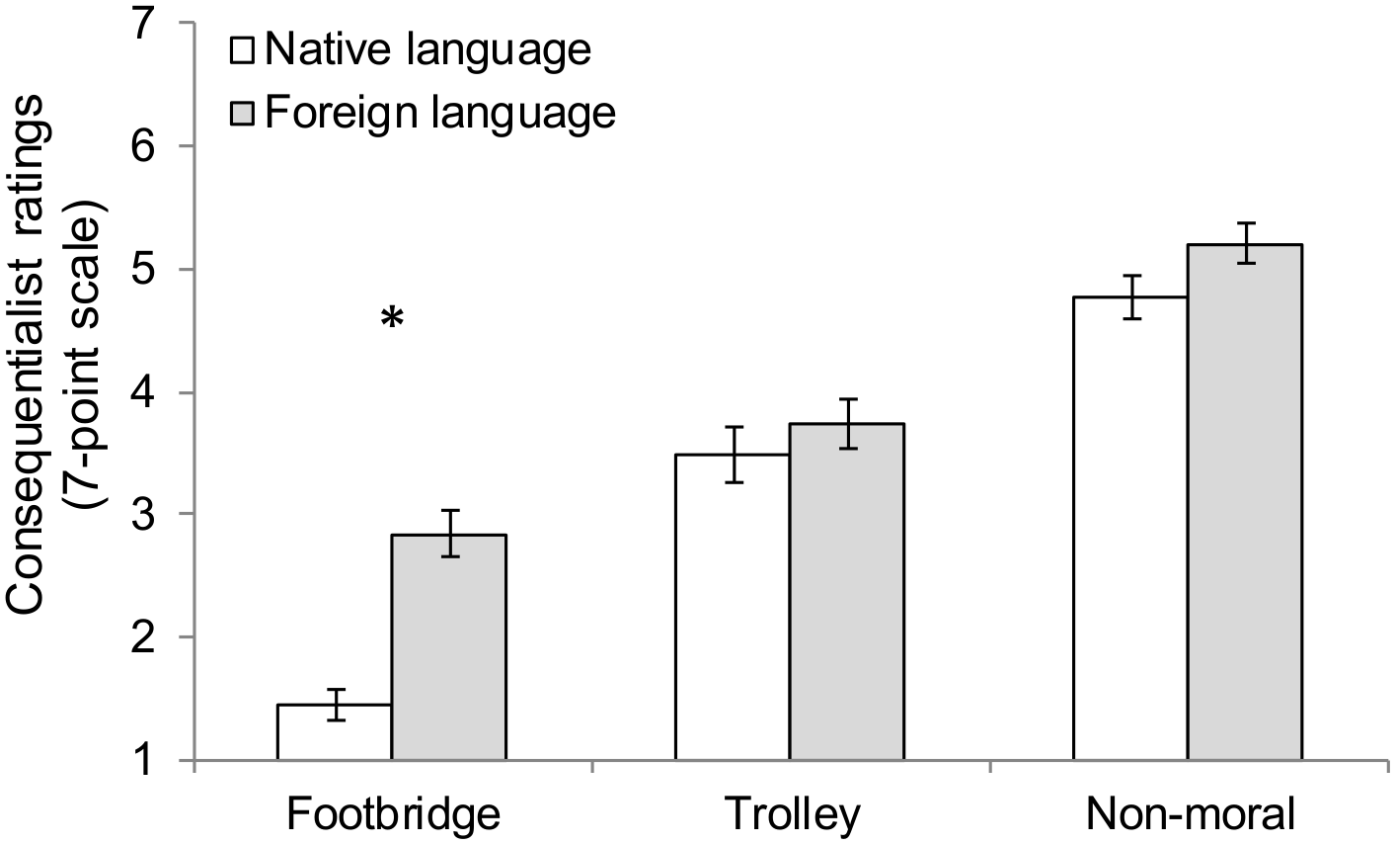
Mean consequentialist ratings in Study 2. Mean consequentialist ratings (1 = *the action is forbidden*, 4 = *the action is permissible*, 7 = *the action is obligatory*) by moral dilemma and language condition (native language = Chinese, foreign language = English). Error bars indicate the standard error of the mean. *p < .05.

The main findings are illustrated in Fig 3. We submitted the permissibility ratings to a 2 (Language: foreign vs. native) × 3 (Dilemma type: footbridge vs. trolley vs. non-moral) mixed-factor analysis of variance (ANOVA) with repeated measures on dilemma type. This ANOVA revealed a main effect of language condition, *F*(1, 149) = 16.80, *p* < .001, η_p_^2^ = .10. As anticipated, mean consequentialist ratings were higher in the foreign language condition (*M*_FL_ = 3.93, CI [3.71, 4.14]) than in the native language condition (*M*_NL_ = 3.23, CI [2.97, 3.49]). Importantly, this main effect was qualified by a significant language × dilemma interaction, *F*(1.92, 286.45) = 5.93, *p* = .003, η_p_^2^ = .04. Mauchly’s test indicated that the assumption of sphericity had been violated, χ^2^ (2, *N* = 151) = 6.09, *p* = .048, therefore degrees of freedom were corrected using Greenhouse-Geisser estimates of sphericity (ε = .96).

The language effect was present only in the footbridge dilemma (*M*_FL_ = 2.84, CI [2.46, 3.22] vs. *M*_NL_ = 1.44, CI [1.21, 1.74]), *t*(143) = 6.09, *p* < .001, *d* = 0.95, Cl [0.95, 1.86]. There was also a main effect of dilemma type, *F*(1.92, 286.45) = 125.01, *p* < .001, η_p_^2^ = .46. In line with previous research, mean consequentialist ratings were lower in the footbridge dilemma (*M* = 2.14, CI [1.89, 2.39]) than in the trolley dilemma (*M* = 3.61, CI [3.31, 3.91]). (For the non-moral dilemma: *M* = 4.99, CI [4.75, 5.23]).

#### Correlations between proficiency and moral judgments

As in Study 1, we created a proficiency score by aggregating a participant’s self-ratings in reading and understanding. We computed correlations between language proficiency and action endorsements (*Yes* = 1, *No* = 0) and between language proficiency and action permissibility ratings (1–7). For the footbridge dilemma, we found that the lower the language proficiency the higher the action endorsements, *r*(150) = –.21, *p* = .009, as well as the higher the permissibility ratings, *r*(149) = –.48, *p* < .001. For the trolley dilemma, there was no correlation between proficiency and action endorsements, *r*(150) = –.02, *p* = .810, or permissibility ratings, *r*(149) = –.14, *p* = .099. For the non-moral dilemma, there was also no correlation between proficiency and action endorsements, *r*(150) = .09, *p* = .288, or permissibility ratings, *r*(149) = –.11, *p* = .170.

#### Distress ratings

The three distress scales (upset, worried, and sad) were highly correlated (Cronbach’s α was .80 for the trolley dilemma, and .81 for the footbridge dilemma). Therefore, we collapsed them into a single distress index by calculating the mean over the three scales. With respect to the native language, the foreign language attenuated distress ratings for *both* moral dilemmas. A 2 (Language: foreign vs. native) × 2 (Dilemma type: trolley vs. footbridge) mixed-factor ANOVA, revealed a significant main effect of language, *F*(1, 150) = 5.04, *p* = .026, η_p_^2^ = .03, which was not qualified by an interaction, *F*(1, 150) = 0.04, *p* = .846, η_p_^2^ < .01. Overall, distress ratings were lower in the foreign language condition (*M*_FL_ = 4.88, 95% CI [4.60, 5.16]) than in the native language condition (*M*_NL_ = 5.38, CI [5.04, 5.72]). Distress ratings were higher in the trolley dilemma (*M* = 5.30, CI [5.06, 5.54]) than in the footbridge dilemma (*M* = 4.96, CI [4.70, 5.23]), as revealed by a significant main effect of dilemma type, *F*(1, 150) = 8.19, *p* = .005, η_p_^2^ = .05. Although one might expect that the footbridge dilemma evokes stronger emotional reactions than the trolley dilemma [16], several studies have failed to find such evidence [31–33].

#### Mediation analysis

We also conducted a mediation analysis to examine whether emotion mediates the effect of language on moral judgment in the footbridge dilemma. (We did not conduct a similar analysis for the trolley dilemma, as no language effect was detected for this item.) We used the SOBEL macro for SPSS [34] and the non-parametric bootstrapping procedure (5000 bootstrapped re–samples). The predictor was language (foreign vs. native) and the mediator distress ratings. The total and indirect effects of language on moral judgment were respectively, 1.41 (*p* < .001) and 1.40 (*p* < .001). An examination of the total indirect effect of language on moral judgment through distress indicated no mediation, since its 95% BCa bootstrap CI contains zero, –0.085 to 0.109.

### Discussion

Study 2 successfully extended the foreign language effect on moral judgment to late Chinese–English bilinguals. It also investigated whether this effect is underpinned by an attenuation of emotions, as it was proposed, but not tested, in previous studies. The use of a foreign language attenuated emotions in *both* moral dilemmas, and the emotion attenuation did not mediate the association between language and moral judgment.

## Study 3

Following Greene’s original proposal [2], one could conjecture that the foreign language effect on moral judgment is found in *personal* dilemmas, such as the footbridge scenario, but not in *impersonal* dilemmas, such as the trolley scenario. The two types of dilemmas vary on a number of dimensions, any one of which could be critical for observing the effect. For example, personal dilemmas involve harm caused using personal force, and the instrumental use of a person [1]. The main aim of Study 3 was to investigate whether the foreign language effect is linked to the personal-impersonal distinction. To this end, we examined an additional personal dilemma (high-emotion), the so-called 'crying baby scenario', and an additional impersonal dilemma (low-emotion), the 'lost wallet scenario' (for an assessment of these dilemmas in terms of emotionality, see [16]). Research consistently reports that the crying baby dilemma is highly emotional, even more than the footbridge dilemma or the trolley dilemma [31]. A further aim was to replicate the findings of Studies 1 and 2 as well as of previous studies showing a foreign language effect in the footbridge dilemma and no effect in the trolley dilemma. To test the robustness of the foreign language effect, we examined yet a different sample of bilinguals: native German speakers who learned English as a foreign language.

### Method

#### Participants

The total sample size was determined in the same way as in Study 2. The a–priori sample size calculation indicated a minimum sample size of 56. Initially, we planned to conduct the entire study at the end of a class session at the Department of Psychology of the Free University Berlin. Forty–eight participants volunteered to participate, but 10 participants were excluded from the analyses as they were not native German speakers. Therefore, we decided to increase the sample size by conducting an online version of the study. No interim analyses or stopping rules were applied.

Seventy-two native German speakers participated in this study (55 female, 15 male, 1 unknown; mean age = 26.63 years, age range = 18–70 years). Thirty-eight participated at the beginning of a lecture at the Free University of Berlin, while 34 in an online version of the study. The online-study participants were recruited from the University Osnabrück and Humboldt University Berlin via email lists. The link to the online survey was active for one week. Within the class and online parts of the study, participants were randomly assigned either to the foreign language condition (*n* = 38; English) or to the native language condition (*n* = 34; German). The level of qualification in English of the participants assigned to the foreign language condition ranged from beginner (A = *basic user*) to advanced (C = *proficient user*), with the majority holding an intermediate level qualification (B = *independent user*) (based on the CEFR; [25]). On average, the participants assigned to the foreign language condition began English education at age 9.02, CI [8.45, 9.60]. These participants were also asked to rate their proficiency in English in terms of conversational fluency, reading, writing, and understanding, on a 5-point scale (1 = *almost none*, 2 = *poor*, 3 = *fair*, 4 = *good*, 5 = *very good*). Averaging across the four measures (Cronbach’s α = .89) they rated their skills as *good* (*M* = 4.05,CI [3.80, 4.26]).

#### Materials and procedure

Participants were presented with the trolley, footbridge, and non-moral dilemma of Studies 1 and 2 along with an extra dilemma of each type (all dilemmas were adapted from [2]; for the full text see Appendix). The new dilemmas included a personal dilemma (crying baby), an impersonal dilemma (lost wallet), and a non-moral scenario. In the crying baby dilemma one must decide whether to smother one’s own child in order to save oneself and several others from being found and killed by enemy soldiers. In the lost wallet dilemma a person in need must decide whether to return a wallet full of cash that seems to belong to a wealthy individual. In the new non-moral dilemma one must decide whether to make two trips home by car rather than a single trip to carry some plants in order to avoid ruining the car’s upholstery. About half of the participants in each language condition received the six dilemmas in a randomized order, while the other half received them in the inverse order. Following each dilemma, participants had to rate the permissibility of the described action on a 7-point scale (1 = *forbidden*, 4 = *permissible*, 7 = *obligatory*; [6]).

### Results

Preliminary analyses revealed no effect of method of administration (in classroom vs. online), or presentation order. Hence, we dropped these factors from subsequent analyses. In contrast to Studies 1 and 2, preliminary analyses revealed a significant gender effect. In line with prior work [13], male participants rated the consequentialist actions as more permissible (*M* = 3.00, CI [2.60, 3.40]) than female participants (*M* = 2.44, CI [2.23, 2.65]), *F*(1, 68) = 6.24, *p* = .015, η_p_^2^ = .08. Given that gender differences were not a focus of the present study, in the analyses below we included gender as a covariate.

#### Moral judgments

**Fig 4:**
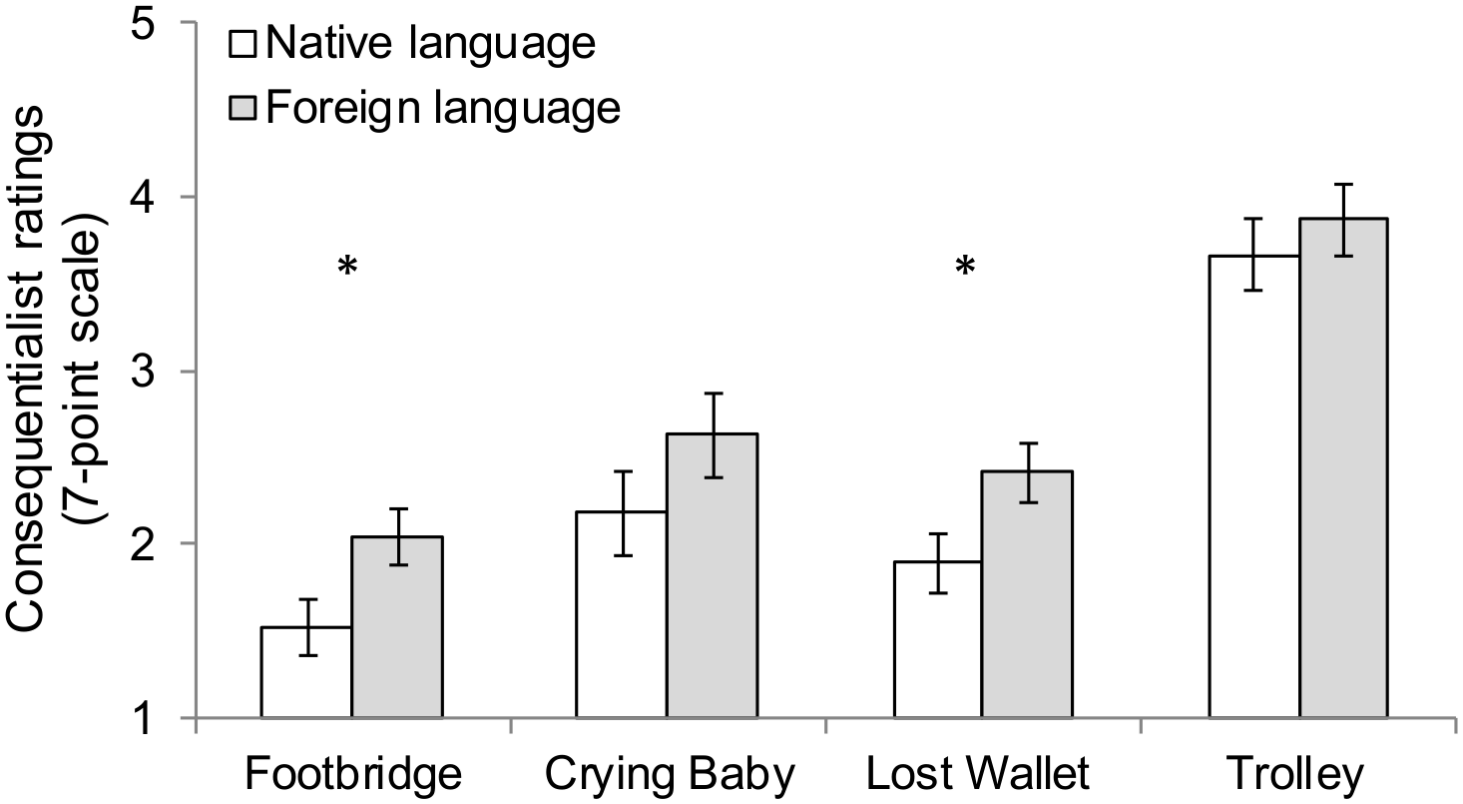
Mean consequentialist ratings in Study 3. Mean consequentialist ratings (1 = *forbidden*, 4 = *permissible*, 7 = *obligatory*) by dilemma type and language condition (native language = German; foreign language = English). Error bars indicate the standard error of the mean. *p < .05.

Two data points were detected as outliers as their values were greater than three standard deviations from the means. We winsorized these two values by aggregating the mean and two standard deviations. The effect of language reported below remains even if we include the original values. The main findings for the moral dilemmas are illustrated in Fig 4. Notice that the pattern of the means of the four dilemmas (from lowest to highest: footbridge, lost wallet, crying baby, trolley) is consistent with that reported in prior research [2]. We conducted a 2 (Language: foreign vs. native) × 4 (Moral dilemmas: 1–4) mixed-factor analysis of covariance (ANCOVA), with gender as a covariate. There was a main effect of language, *F*(1, 68) = 6.29, *p* = .015, η_p_^2^ = .09. Mean consequentialist ratings were higher in the foreign language condition (*M*_FL_ = 2.74, CI [2.51, 2.97]) than in the native language condition (*M*_NL_ = 2.32, CI [2.08, 2.57]). This effect was not qualified by a language × dilemma interaction, *F*(2.63, 178.89) = 0.29, *p* = .811, η_p_^2^ < .01. There was no main effect of dilemma, *F*(2.63, 178.89) = 2.58, *p* = .063, η_p_^2^ = .04. Mauchly’s test indicated that the assumption of sphericity had been violated, χ^2^ (5, *N* = 71) = 16.53, *p* = .005, therefore degrees of freedom were corrected using Greenhouse-Geisser estimates of sphericity (ε = .88).

A similar analyses performed for the non-moral dilemmas showed no significant differences between the foreign language condition (*M* = 4.87, CI [4.60, 5.13]) and the native language condition (*M* = 4.50, CI [4.22, 4.78]), *F*(1, 70) = 3.60, *p* = .062, *f* = .22. There was a main effect of dilemma, *F*(1, 70) = 79.85, *p* < .001, *f* = 1.07, but no language × dilemma interaction, *F*(1, 70) = 0.55, *p* = .461, *f* = .09.

In order to examine whether the foreign language effect is present in the footbridge dilemma and absent in the trolley dilemma, we conducted planned comparisons using multivariate analysis of covariance (MANCOVAs) with gender as a covariate. Confirming the pattern found in the previous studies, we detected a significant foreign language effect in the footbridge dilemma (*M*_FL_ = 2.04, CI [1.73, 2.36], *M*_NL_ = 1.53, CI [1.20, 1.87]), *F*(1, 68) = 4.79, *p* = .032, η_p_^2^ = .07, but not in the trolley dilemma (*M*_FL_ = 3.87, CI [3.45, 4.29], *M*_NL_ = 3.67, CI [3.21, 4.12]), *F*(1, 68) = 0.42, *p* = .517, η_p_^2^ = .01. We also performed similar analyses for the new dilemmas. A significant foreign language effect was observed in the lost wallet dilemma (*M*_FL_ = 2.42, CI [2.08, 2.76], *M*_NL_ = 1.90, CI [1.54, 2.27]), *F*(1, 68) = 4.29, *p* = .042, η_p_^2^ = .06, but not in the crying baby dilemma (*M*_FL_ = 2.63, CI [2.15, 3.11], *M*_NL_ = 2.18, CI [1.67, 2.70]), *F*(1, 68) = 1.60, *p* = .211, η_p_^2^ = .02.

#### Correlations between proficiency and moral judgment

Preliminary analyses showed that the participants in the foreign language condition reported high self-ratings of proficiency in reading and understanding (*M* = 8.61, CI [8.12, 9.08]). As a result, there was little variation in proficiency and thus weak associations between proficiency and moral judgment. The only dilemma for which we observed a significant (negative) correlation was the lost wallet: the lower the language proficiency, the higher the consequentialist ratings, *r*(70) = –.30, *p* = .011.

### Discussion

In Study 3 we examined whether the foreign language effect is linked to the personal-impersonal distinction. The results do not support this hypothesis. We observed a foreign language effect, but this effect was not qualified by an interaction with dilemma type. Replicating the findings of Studies 1 and 2, detailed analyses showed that foreign language influenced moral judgments in the footbridge (personal) but not in the trolley (impersonal) dilemmas. Critically, the effect was also present in an impersonal dilemma (lost wallet). A surprising finding was that the foreign language effect was absent in the crying baby dilemma. This could be because, in contrast to the other dilemmas, in this dilemma the victim of the action would die regardless. In economic jargon, performing the action is the dominant option, because its payoff is better than the payoff of omitting the action. Participants in both language conditions might have thought about the dominance relation, which would explain both the high permissibility ratings (which are consistent with previous research [2]) and the absence of a foreign language effect. We wish to thank an anonymous reviewer for pointing to us this explanation.

## Meta-analyses

In Studies 1 and 3 there was a trend towards a foreign language effect in the trolley dilemma. We examined this possibility by performing a random effects meta-analysis summarizing the three studies. For completion, we also performed a similar meta-analysis for the footbridge dilemma.

### Trolley dilemma

**Fig 5:**
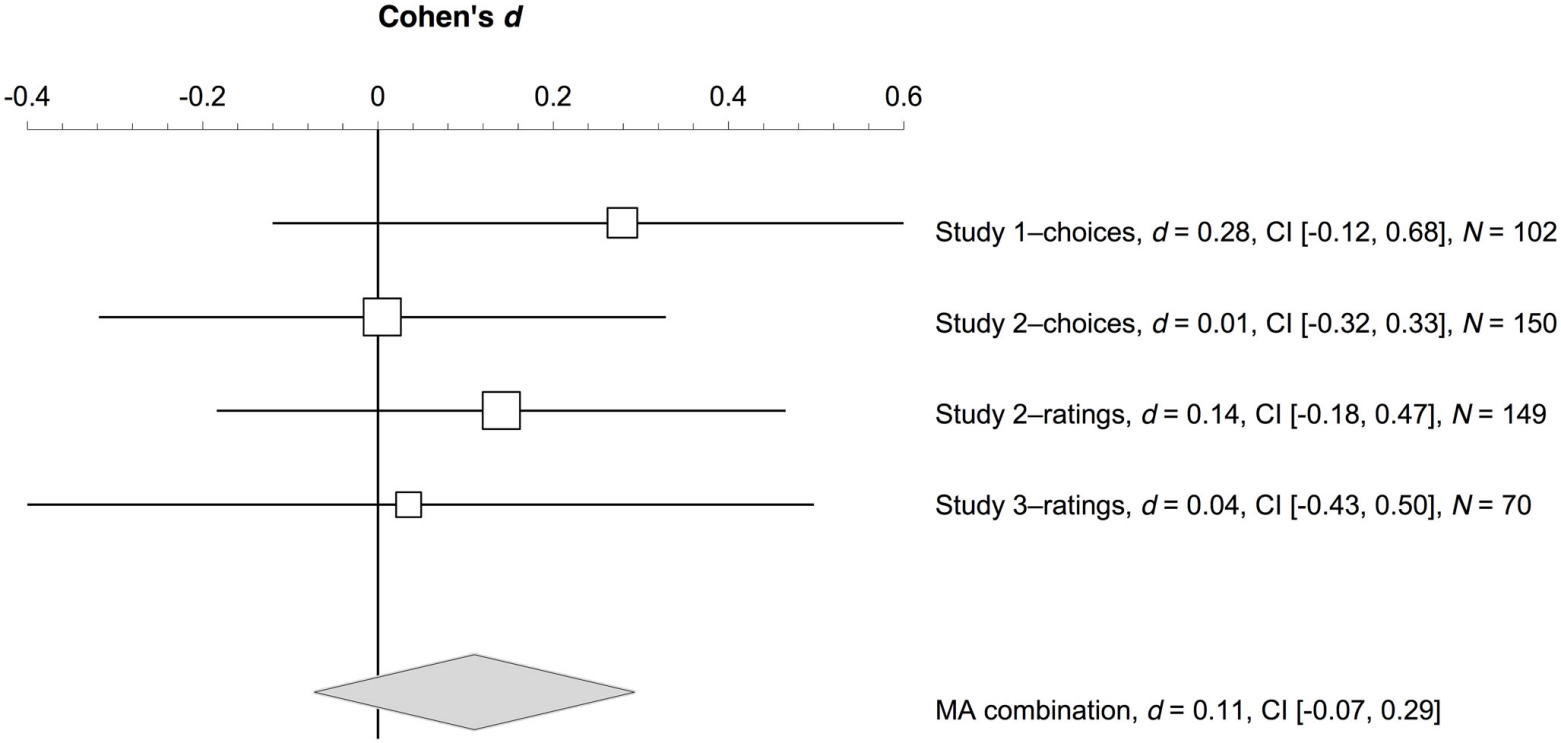
Forest plot. Forest plot indicating the effect sizes (squares) and 95% CIs (lines) of the three studies for the foreign language effect on the trolley dilemma. The diamond illustrates the overall effect size and its 95% CI given by a random effects meta-analysis (MA) that combines the three studies.

Fig 5 illustrates the results of a random effects meta-analysis summarizing the three studies regarding the moral choices and judgments in the trolley dilemma (we used *Exploratory Software for Confidence Intervals* [35]). The main result of each study (foreign language effect) is represented as a square marking the effect size (Cohen’s *d*), and its 95% CI. The diamond represents the overall effect size of the meta-analysis, and its 95% CI. The overall effect size was 0.11, 95% CI [–0.07, 0.29], which is interpreted as *no effect* [36]. In conclusion, there was no foreign language effect for the trolley dilemma across the three studies.

### Footbridge dilemma

**Fig 6:**
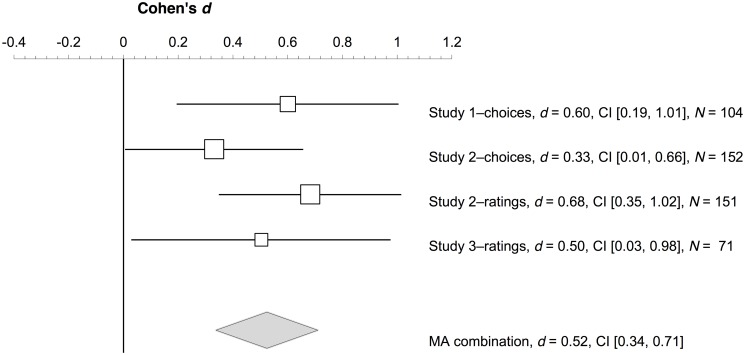
Forest plot. Forest plot indicating the effect sizes (squares) and 95% CIs (lines) of the three studies for the foreign language effect on the footbridge dilemma. The diamond illustrates the overall effect size and its 95% CI given by a random effects meta-analysis (MA) that combines the three studies.

Fig 6 illustrates the results of a random effects meta-analysis summarizing the three studies regarding the moral choices and judgments for the footbridge dilemma between the foreign language and the native language conditions. The overall effect size was 0.52, 95% CI [0.34, 0.71], which is interpreted as *intermediate effect* [36]. In conclusion, there was an intermediate foreign language effect for the footbridge dilemma across the three studies.

## General Discussion

The present studies provide strong evidence that the use of a foreign language influences the moral evaluation of complex moral dilemmas. In line with previous work [19, 20], foreign language increased the rate of consequentialist responses in the footbridge dilemma but not in the trolley dilemma (Studies 1–3, meta-analyses). However, in contrast to previous theorizing, the results do not support the claim that this effect is driven by reduced emotionality. Foreign language attenuated emotions in response to *both* the footbridge and trolley dilemmas, and this emotion attenuation did not mediate the effect of foreign language on moral judgment (Study 2). Furthermore, the foreign language effect was not constrained to personal dilemmas; it was also present in an impersonal dilemma (lost wallet; Study 3). Interestingly, the foreign language effect was absent in the crying baby dilemma, which we suggested might be due to that this dilemma had a distinctive feature that was absent in all other moral dilemmas: the negative outcome of the action (the baby’s death) would occur anyway, even if the action were not performed.

If the emotional attenuation is not a viable explanation for the foreign language effect on moral judgment then what drives this effect? Why was the effect absent from the trolley dilemma but present in the footbridge and lost wallet dilemmas? Perhaps the critical difference is that the trolley dilemma does not involve a “taboo” or prohibited action. Social and moral rules prohibit us from pushing people or keeping lost wallets. However, we have no general rules prohibiting flipping switches (see Cushman’s dual-system framework of morality [37]). We propose that foreign language may influence moral judgment by reducing the mental accessibility of social and moral rules. For this explanation to work, one has to assume that in the case of flipping the switch the categorization of the action at a more abstract level (killing the collateral innocent victim) is less automatic than the categorization of the other actions, such as keeping the wallet as a prototypical form of stealing.

Evidence that foreign language reduces the accessibility of social and moral rules comes from a study showing that foreign language promotes less condemnation of violations of everyday social and moral norms, such as cutting in line when in a hurry, or cheating in an exam [38]. Further evidence comes from a study in which participants were asked to translate swearwords either from a native to a foreign language or vice versa [39]. In the native to foreign language translations, participants used “stronger” words to translate politically incorrect swearwords which were directed against social groups, compared to other types of swearwords. The authors of this study argued that in a foreign language the social and cultural norms are less salient, which makes it easier for people to use inappropriate swearwords (for similar views see [29, 40]).

One way through which foreign language might reduce the activation of social and moral norms, is by limiting access to relevant autobiographical memories. Research suggests that memories are language specific, and therefore are more accessible when the language used at retrieval matches the one present at encoding [41–43], that is, the native language. Research suggests that several moral and social rules are learned through social communication [44], and that a great chunk of such rules concern prohibitions of specific actions [45]. An analysis of a large corpus of data demonstrated that 99% of child-directed speech about rules of conduct, referred to the prohibition of particular actions, such as “Don’t throw paper on the floor!”, but there were also many cases of parents just saying “No!” [45]. A foreign language might evoke memories related to such prohibitions to a lesser extent than the native language. Consistent with this claim, Harris and colleagues [46, 21] showed that the use of a foreign language reduced electrodermal activity in response to childhood reprimands (“Don’t do that!”).

One limitation of the present research is that we employed a restricted number of moral dilemmas. Future research should examine a wider variety of moral scenarios [47, 48, 28]. A second limitation is that some of the scenarios we used (e.g., footbridge, trolley) are arguably “exotic” and distant from real life [49, 50]. Notice, however, that the foreign language effect extended to the more mundane lost wallet scenario. It would be worth investigating whether the effect generalizes to other realistic situations (see [38]) and actual behavior. A third limitation is that we measured emotional reactions by means of rating scales *after* the moral judgment was made. It could be that the higher emotion ratings in the trolley dilemma, as compared to the footbridge dilemma, are related to post-decisional processes. But note that previous studies have also measured emotions *before* the moral judgment was made and they failed to find support for the claim that the footbridge dilemma evokes more negative emotion than the trolley dilemma ([31]; see also [32]). Please note that we do not wish to claim that foreign language does not influence affect, Study 2 demonstrated that it does [23]. Furthermore, studies in the domain of risk and benefit perception have shown a foreign language effect that is mediated by affect [51]. Rather, the claim is that the foreign language effect on moral dilemmas might be more complex. A fourth limitation is that the increase in consequentialist responses found in the foreign language condition might be because participants assigned to that condition might have assumed that the situations involved foreign people in a foreign country. Research suggests that feelings of social connection to the characters involved in a dilemma influences moral evaluations [52–54]. But notice that the effect was present also in a study where participants were explicitly instructed to assume that the characters were co-nationals and that the situation took place in their country [38].

The present findings have important societal implications. International decisions such as those taken by the Economic European Community and the United Nations often involve communication in a foreign language (mostly in English). A number of such decisions involve a tradeoff between causing intentional harm to a number of individuals in the near future (e.g., by imposing strict economic rules), to increase the prosperity of a greater number of individuals in a relatively more distant future. If the use of foreign language reduces access to knowledge of social norms and deontological moral principles, then international decisions may be swayed (for better or worse) towards a consequentialist choice.

## Appendix

### Dilemmas used in Studies 1, 2, and 3 (English versions)

#### Personal dilemmas

**Footbridge:** A runaway trolley is heading down the tracks toward five workmen who will be killed if the trolley proceeds on its present course. You are on a footbridge over the tracks, in between the approaching trolley and the five workmen. Next to you on this footbridge is a stranger who happens to be very large. The only way to save the lives of the five workmen is to push this stranger off the bridge and onto the tracks below where his large body will stop the trolley. The stranger will die if you do this, but the five workmen will be saved. Is it appropriate for you to push the stranger on to the tracks in order to save the five workmen? [This dilemma was used in all studies]

**Crying Baby:** Enemy soldiers have taken over your village. They have orders to kill all remaining civilians. You and some of your townspeople have sought refuge in the cellar of a large house. Outside you hear the voices of soldiers who have come to search the house for valuables. Your baby begins to cry loudly. You cover his mouth to block the sound. If you remove your hand from his mouth his crying will summon the attention of the soldiers who will kill you, your child, and the others hiding out in the cellar. To save yourself and the others you must smother your child to death. Is it appropriate for you to smother the child in order to save yourself and the other townspeople? [This dilemma was used in Study 3]

#### Impersonal dilemmas

**Trolley:** You are at the wheel of a runaway trolley quickly approaching a fork in the tracks. On the tracks extending to the left is a group of five railway workmen. On the tracks extending to the right is a single railway workman. If you do nothing the trolley will proceed to the left, causing the deaths of the five workmen. The only way to avoid the deaths of these workmen is to hit a switch on your dashboard that will cause the trolley to proceed to the right, causing the death of the single workman. Is it appropriate for you to hit the switch in order to save the lives of the five workmen? [This dilemma was used in all studies]

**Lost Wallet:** You are walking down the street when you come across a wallet lying on the ground. You open the wallet and find that it contains several hundred euros in cash as well the owner's driver's license. From the credit cards and other items in the wallet it's very clear that the wallet's owner is wealthy. You, on the other hand, have been hit by hard times recently and could really use some extra money. You consider sending the wallet back to the owner without the cash, keeping the cash for yourself. Is it appropriate for you to keep the money you found in the wallet in order to have more money for yourself? [This dilemma was used in Study 3]

#### Non-moral dilemmas

**Train or Bus:** You need to travel from Bologna [*Beijing*; *Berlin*] to Ancona [*Jinan*; *Leipzig*] in order to attend a meeting that starts at 2:00 PM. You can take either the train or the bus. The train will get you there just in time for your meeting no matter what. The bus is scheduled to arrive an hour before your meeting, but the bus is occasionally several hours late because of traffic. It would be nice to have an extra hour before the meeting, but you cannot afford to be late. Is it appropriate for you to take the train instead of the bus in order to ensure you are not being late for your meeting? [This dilemma was used in all studies]

**Plant Transport:** You are bringing home a number of plants from a store that is about 5 kilometers from your home. The trunk of your car, which you've lined with plastic to catch the mud from the plants, will hold most of the plants you've purchased. Is it appropriate for you to make two trips home in order to avoid ruining the upholstery of your car? [This dilemma was used in Study 3]

## Supporting Information

S1 Dataset Dataset of Study 1. (ZIP)

S2 Dataset Dataset of Study 2. (ZIP)

S3 Dataset Dataset of Study 3. (ZIP)
